# Limited efficacy of temozolomide alone for astrocytoma, IDH-mutant, CNS WHO grades 2 or 3

**DOI:** 10.1007/s11060-022-04128-y

**Published:** 2022-09-16

**Authors:** Jonathan Weller, Sophie Katzendobler, Jens Blobner, Frederic Thiele, Hannes Becker, Stefanie Quach, Rupert Egensperger, Maximilian Niyazi, Bogdana Suchorska, Niklas Thon, Michael Weller, Joerg-Christian Tonn

**Affiliations:** 1 Department of Neurosurgery, Medical Center of the University of Munich, Marchioninistrasse 15, 81377 Munich, Germany; 2 Department of Neurosurgery, University Hospital of Tubingen, Tübingen, Germany; 3 Center for Neuropathology and Prion Research, Medical Center of the University of Munich, Munich, Germany; 4 Department of Radiotherapy and Radiation Oncology, Medical Center of the University of Munich, Munich, Germany; 5 Department of Neurosurgery, Sana Hospital Duisburg, Duisburg, Germany; 6 Department of Neurology, University Hospital and University of Zurich, Zurich, Switzerland; 7 German Cancer Consortium (DKTK), Partner Site Munich, Munich, Germany

**Keywords:** Astrocytoma, Temozolomide, Radiotherapy, Hypermutation

## Abstract

**Purpose:**

The role of temozolomide chemotherapy alone in isocitrate dehydrogenase (IDH)-mutant astrocytomas has not been conclusively determined. Radiotherapy might be superior to temozolomide. Recent studies have linked temozolomide with induction of hypermutation and poor clinical course in some IDH-mutant gliomas.

**Methods:**

In this retrospective study, 183 patients with astrocytoma, IDH*-*mutant, CNS WHO grade 2 or 3 and diagnosed between 2001 and 2019 were included. Patients initially monitored by wait-and-scan strategies or treated with radiotherapy or temozolomide alone were studied. Patient data were correlated with outcome. Matched pair and subgroup analyses were conducted.

**Results:**

Radiotherapy was associated with longer progression-free survival than temozolomide (6.2 vs 3.4 years, *p* = 0.02) and wait-and-scan strategies (6.2 vs 4 years, *p* = 0.03). Patients treated with radiotherapy lived longer than patients treated with temozolomide (14.4 vs 10.7 years, *p* = 0.02). Survival was longer in the wait-and-scan cohort than in the temozolomide cohort (not reached vs 10.7 years, *p* < 0.01). Patients from the wait-and-scan cohort receiving temozolomide at first progression had significantly shorter survival times than patients treated with any other therapy at first progression (p < 0.01). Post-surgical T2 tumor volume, contrast enhancement on MRI and WHO grade were associated with overall survival in univariate analyses (p < 0.01).

**Conclusion:**

The results suggest superiority of radiotherapy over temozolomide and wait-and-scan strategies regarding progression-free survival and superiority of radiotherapy over temozolomide regarding overall survival. Our results are consistent with the notion that early temozolomide might compromise outcome in some patients.

## Introduction

Patients with IDH-mutant (IDHmut) WHO grade 2 or 3 astrocytomas often show a long clinical course and are subjected to a multitude of therapeutic interventions over time. This poses a challenge to health care professionals, patients and caregivers as to determine time point and modality of tumor specific treatment. Adverse effects from therapy need to be weighed against tumor control. Gold standard for IDHmut astrocytomas is initial maximum safe resection [1]. Postoperative radiotherapy (RT) followed by procarbazine, CCNU and vincristine (PCV) in WHO grade 2 astrocytomas or maintenance temozolomide (TMZ) in WHO grade 3 astrocytomas should be initiated in high-risk patients defined as older than 40–45 years of age, after incomplete resection or with neurological deficits beyond seizures [1–3]. Of note, predictive and prognostic implication of grading in IDHmut gliomas remains controversial since data from large cohorts showed only marginal differences in overall survival between WHO grade 2 and 3 [4].

The role of surveillance strategies versus RT alone, PCV alone, TMZ alone or combined modality treatment in the treatment of IDHmut gliomas has not been conclusively determined. Surveillance strategies can be pursued in younger patients and after gross total resection [5]. Early RT as opposed to RT at first progression was shown to be associated with prolonged progression-free survival, but not overall survival in WHO grade 2 gliomas [6]. A major concern of early RT in young patients is the potential long-term neurocognitive decline as a complication of treatment [7, 8]. The role of chemotherapy alone remains investigational, and data are scarce. Guidelines give leeway for chemotherapy regimens, omitting RT, with either PCV or TMZ if the tumor volume is large, the patient is young, or RT is not indicated for other reasons [1]. In high-risk WHO grade 2 IDHmut astrocytomas, TMZ monotherapy was associated with shorter progression-free survival than RT alone [9]. Similar outcome of chemotherapy alone versus RT has been reported in WHO grade 3 gliomas with a possible superiority of PCV over TMZ [10, 11]. The CODEL study (NCT00887146) was closed and redesigned prematurely due to inferior outcome of patients with WHO grade 3 oligodendroglioma treated with TMZ, as compared with the other study groups of RT alone or RT with concomitant and maintenance TMZ [12]. A retrospective analysis failed to demonstrate benefit for progression-free survival of TMZ alone over resection only or active surveillance after biopsy in WHO grade 2 oligodendrogliomas [13]. In addition to the rather discouraging clinical data on TMZ alone for lower grade gliomas, molecular analyses have reported induction of hypermutation by TMZ therapy in subgroups of IDHmut gliomas [14, 15]. TMZ-driven hypermutational genotypes have been associated with shorter survival times and distant recurrence in initially low grade, IDHmut gliomas [16, 17]. In this context, it is unclear whether TMZ alone is beneficial or even potentially detrimental in the treatment of IDHmut gliomas in the long-term.

Here we set out to investigate the long-term outcome of a consecutive single-center series of 183 patients diagnosed with CNS WHO grade 2 or 3 IDHmut astrocytomas either monitored postoperatively by means of active surveillance or treated with TMZ alone or treated with RT alone.

## Patients and methods

### Patient evaluation

Patients with newly diagnosed *IDH1*- or *IDH2*-mutant astrocytomas without 1p/19q-codeletion diagnosed at the Department of Neurosurgery of the University Hospital Munich between 2001 and 2019 were included in this study. The study was waived by the institutional Ethics committee (project number 21-0612). Patients with histological CNS WHO grade 4 astrocytomas according to WHO 2021 were excluded [18]. Patient-related and clinical parameters as well as complications from therapy, classified according to CTCAE version 5.0 (Common Terminology Criteria for Adverse Events), were assessed (Table 1) [19]. If initial imaging was performed due to symptoms not explicable by the lesion, the diagnosis was termed “incidental”. In patients with multiple infiltrated lobes, the location in Table 1 referred to the most infiltrated lobe. Therapy recommendations were given based on multidisciplinary tumor board decisions and options were discussed with the patients. Besides wait-and-scan (W&S) strategies that had been pursued in many patients with histological WHO grade 2 gliomas at our department, tumor board recommendations often left the option for post-surgical monotherapy strategies.

**Table 1:** Patient-related and clinical characteristics overall and in different cohorts

Parameter	All patients (n = 183)	Wait-and-scan (n = 98)	TMZ (n = 48)	RT (n = 37)	*p* value
Age (years)					
Median	37	35	37	38	0.02*
Range	16–76	16–69	21–76	25–56	
Sex, n (%)					
Female	84 (46)	48 (49)	19 (40)	17 (46)	0.57
Male	99 (54)	50 (51)	29 (60)	20 (54)	
KPS at first admission, n (%)					
100	7 (4)	6 (6)	1 (2)	0 (0)	0.04*
90	149 (81)	82 (84)	34 (71)	33 (89)	
80	27 (15)	10 (10)	13 (27)	4 (11)	
< 80	0 (0)	0 (0)	0 (0)	0 (0)	
Trigger for diagnostic workup, n (%)					
Incidental finding	46 (25)	32 (33)	10 (21)	6 (16)	0.1
Epileptic seizure	103 (56)	51 (52)	24 (50)	27 (73)	
Neurological deficit	35 (19)	15 (15)	14 (29)	4 (11)	
CNS WHO grade, n (%)					
Grade 2	116 (63)	86 (88)	17 (35)	13 (35)	< 0.01*
Grade 3	67 (37)	12 (12)	31 (65)	24 (65)	
Localization, n (%)					
Frontal	92 (50)	46 (47)	24 (50)	22 (60)	0.87
Temporal	55 (30)	34 (35)	16 (33)	5 (14)	
Insular	13 (7)	7 (7)	2 (4)	4 (11)	
Parietal	16 (9)	9 (9)	4 (8)	3 (8)	
Occipital	1 (1)	1 (1)	0 (0)	0 (0)	
Midline	6 (3)	1 (1)	2 (4)	3 (8)	
Laterality, n (%)					
Left	97 (53)	54 (55)	24 (50)	19 (51)	0.67
Right	78 (43)	43 (44)	20 (42)	15 (41)	
Bilateral	8 (4)	1 (1)	4 (8)	3 (8)	
Post-surgical T2 tumor volume (cm^3^)					
Median	22	11	58	16	< 0.01*
Range	0–319	0–200	0–319	0–64	
CE on initial MRI, n (%)	55 (30)	14 (14)	27 (56)	14 (38)	< 0.01*

The right column shows *p*-values when comparing numbers and values of the different cohorts. Statistically significant values, i.e., *p*-values of 0.05 or below (ANOVA and Kruskal–Wallis tests), are depicted with asterisks

*KPS* Karnofsky Performance Status; *CNS* central nervous system; *WHO* World Health Organization; *CE* contrast enhancement; *MRI* magnetic resonance imaging

The date of histological sampling through stereotactic biopsy or tumor resection was set as date of diagnosis. Progression-free survival was defined as the time interval from diagnosis to first progression according to RANO (Response Assessment in Neuro-Oncology) criteria [20]. TMZ was given in a 5/28-schedule and a 150–200 mg/m^2^ dose [1, 9]. In the RT cohort, only patients who had received involved-field RT with 50.4–60 Gy in 1.8–2.0 fractions were included. Overall survival was defined as the time interval between the date of diagnostic surgery and date of death. Patients were monitored through regular visits with up-to-date MRI (magnetic resonance imaging) in the outpatient clinic of the Department of Neurosurgery. Outpatient visits were scheduled every 6 months in case of stable disease and after completion of therapy. When progression was suspected, but RANO criteria had not been met, intervals were 2–3 months. During TMZ, patients without compromising side effects were seen every 2–3 months. Patients treated with RT were first seen 6 weeks after completion of the treatment.

### Histopathology and molecular analyses

Histological samples were classified according to the WHO 2021 classification [18]. Sequencing of sodium bisulfite-modified DNA was performed to determine MGMT promoter (O6-methylguanine-DNA methyltransferase) methylation status. Microsatellite markers were utilized to assess allelic loss on chromosomes 1p and 19q. Assessment of codon 132 and codon 172 for IDH genes 1 and 2 was performed in all patients through pyrosequencing [21, 22].

### Tumor volumes

Initial and postoperative tumor volumes on T2 weighted MRI were manually segmented utilizing Brainlab Elements Smartbrush Software (Brainlab Elements Smartbrush, Munich, GER). In case of tumor resection, patients are subjected to postoperative MRI during inpatient treatment and within 48–72 h after histological sampling at our institution. Matched-pair analyses were conducted to eliminate residual, postoperative tumor volume as a potential confounder. Matching was done manually according to the segmented, postoperative T2 volume and contrast enhancement on MRI, before initiation of chemotherapy or RT. Pairing was accepted if the absolute difference between the volumes did not exceed 10% of the larger volume of the match [13].

### Statistical analysis

Statistical tests were performed and figures designed utilizing GraphPad PRISM software version 9.3.1. Kaplan–Meier estimator method, Mantel–Cox (Log-rank) and Gehan–Breslow–Wilcoxon tests were used for comparison of progression-free and overall survival. Continuous and categorial variables were compared by conventional t-tests, ANOVA and χ^2^ tests respectively. Kruskal–Wallis test was used for comparison of non-parametric, ordinal variables. For univariate analyses, log-rank tests and Cox proportional hazards regression models were used. Associations were considered statistically significant when p-values were 0.05 or below.

## Results

### Study population and clinical parameters

**Fig. 1:**
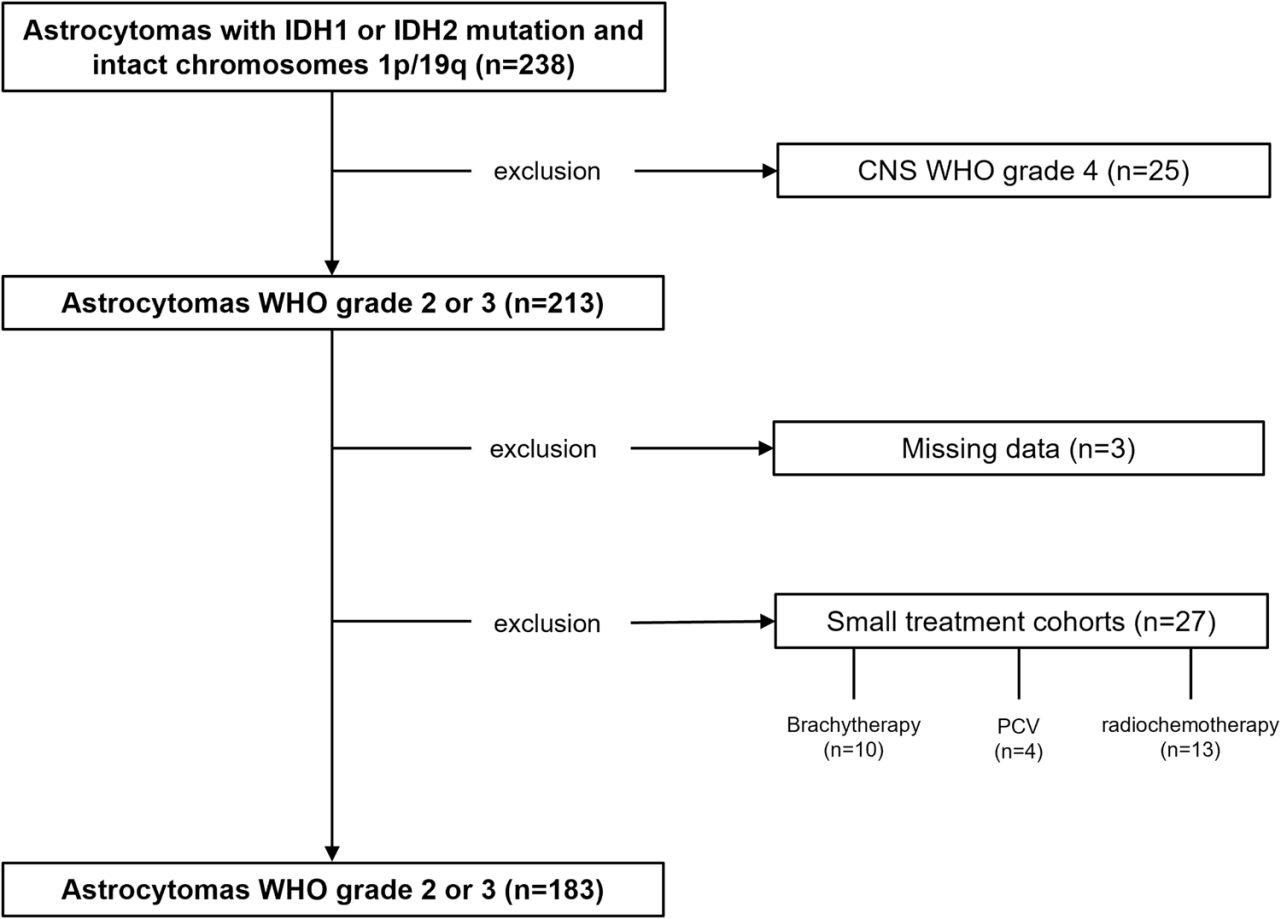
CONSORT figure. Patients having received the diagnosis of an IDH-mutant glioma between the years 2001 and 2019 were stratified according to initial histology. Exclusion criteria were a histological WHO grade 4 at initial diagnosis (n = 25), missing data (n = 3) or small treatment groups (n = 27). *CNS* central nervous system; *WHO* World Health Organisation; *PCV* procarbazine + CCNU + vincristine

Of 213 patients with IDHmut WHO grade 2 or 3 astrocytomas treated at our center between 2001 and 2019, 183 patients could be allocated to three different cohorts according to the post-surgical management strategy: W&S, TMZ alone or RT alone (Fig. 1, Table 1). 30 patients were treated otherwise, e.g., with radiochemotherapy, brachytherapy, photodynamic therapy, or combinations thereof. Initial tumor resection was performed in 100 patients (55%), 83 patients (45%) received a stereotactic biopsy. The median age at diagnosis was 37 years (range 16–76). MGMT promoter methylation was present in 139 patients (76%). Partially methylated promoters were seen in 27 patients (15%). In 17 patients (9%), MGMT promoter methylation was not detected. After biopsy or tumor resection, 98 patients (54%) were not further treated but monitored through regular outpatient visits until progression, 48 patients (26%) received TMZ alone and 37 patients (20%) were treated with RT alone. At the time of database closure, 135 patients (74%) had experienced tumor progression and 33 patients (18%) had died from tumor-related causes. There was no significant difference in sex distribution, trigger for diagnostic workup, tumor location or affected brain hemisphere between the cohorts. Patients were eldest in the RT cohort (*p* = 0.02). The proportion of patients with KPS of 80 was highest in the TMZ cohort (*p* = 0.04). The median postoperative T2 tumor volume was highest in the TMZ cohort (*p* < 0.01). The relative distribution of WHO grade 2 and WHO grade 3 astrocytomas was identical in the RT and TMZ cohort, but the proportion of WHO grade 2 astrocytomas was higher in the W&S cohort (*p* < 0.01) (Table 1). In the TMZ cohort, a median of 6 cycles of chemotherapy was completed (mean and range of cycles completed: 8, 2–15). The mean dose applied to the patients who were initially treated with RT was 60 Gy (range 50.4–60 in fractions of 1.8–2.0). In 2 patients (4%) treated with TMZ, CTCAE grade 3 thrombocytopenia demanded hospitalization. No grade 3 or higher toxicity was reported in the RT cohort.

### Progression-free and overall survival

**Fig. 2:**
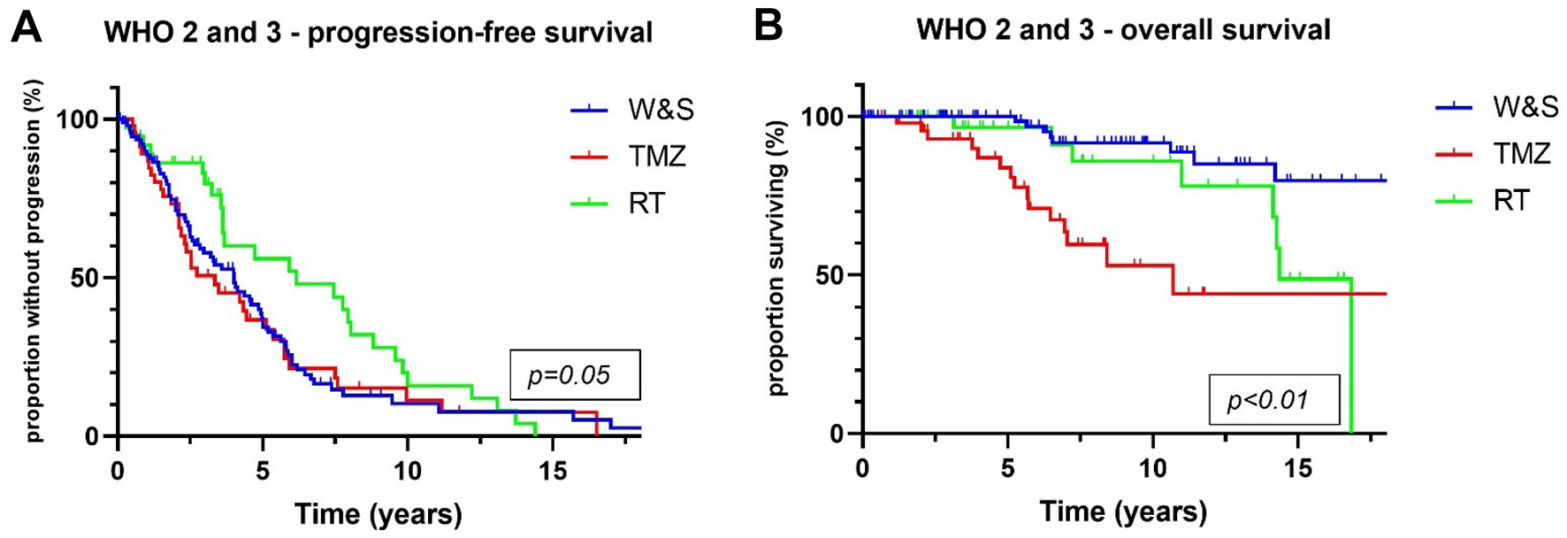
Overall, radiotherapy was associated with longer progression-free survival than wait-and-scan strategies or temozolomide alone (p = 0.05) (**A**). Patients treated with temozolomide monotherapy showed significantly shorter overall survival than patients with other postoperative strategies (p < 0.01) (**B**). WHO World Health Organization; W&S wait-and-scan strategy; TMZ temozolomide; RT radiotherapy

**Fig. 3:**
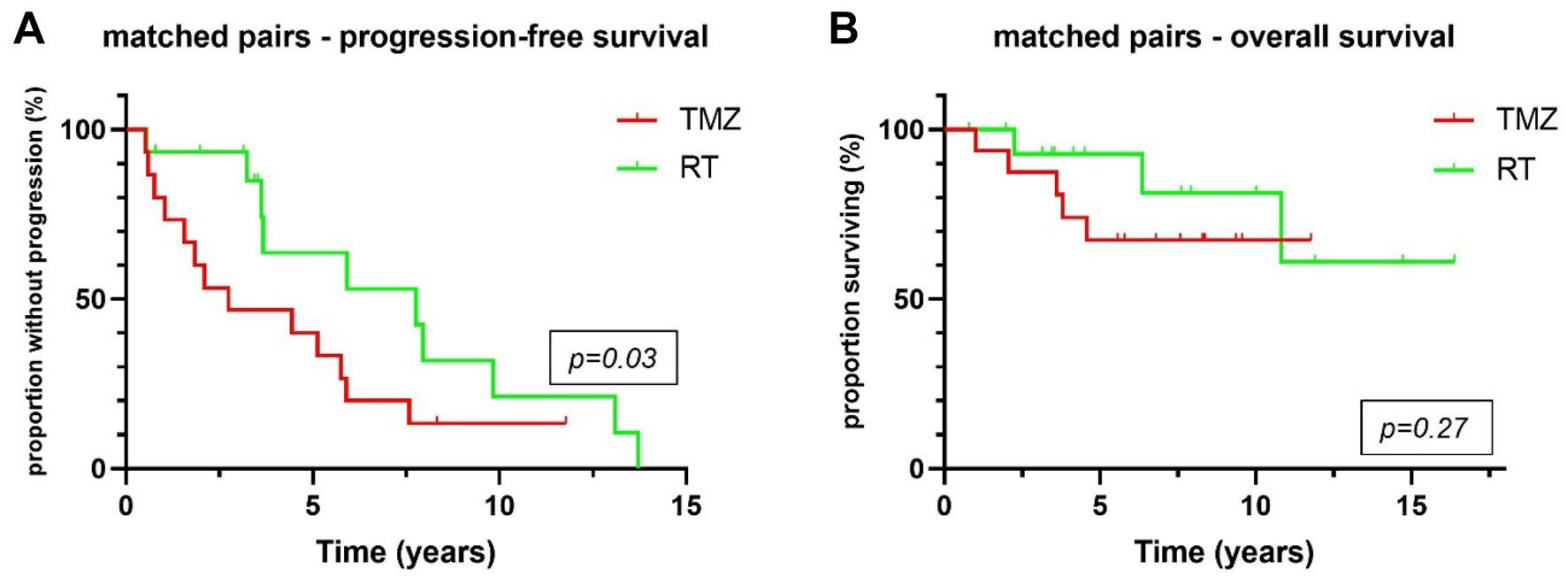
In a matched-pair analysis accounting for post-surgical tumor volume and WHO grade, radiotherapy was superior in terms of progression-free survival (p = 0.03) (**A**), but not overall survival (p = 0.27) (**B**). *TMZ* temozolomide; *RT* radiotherapy

**Fig. 4:**
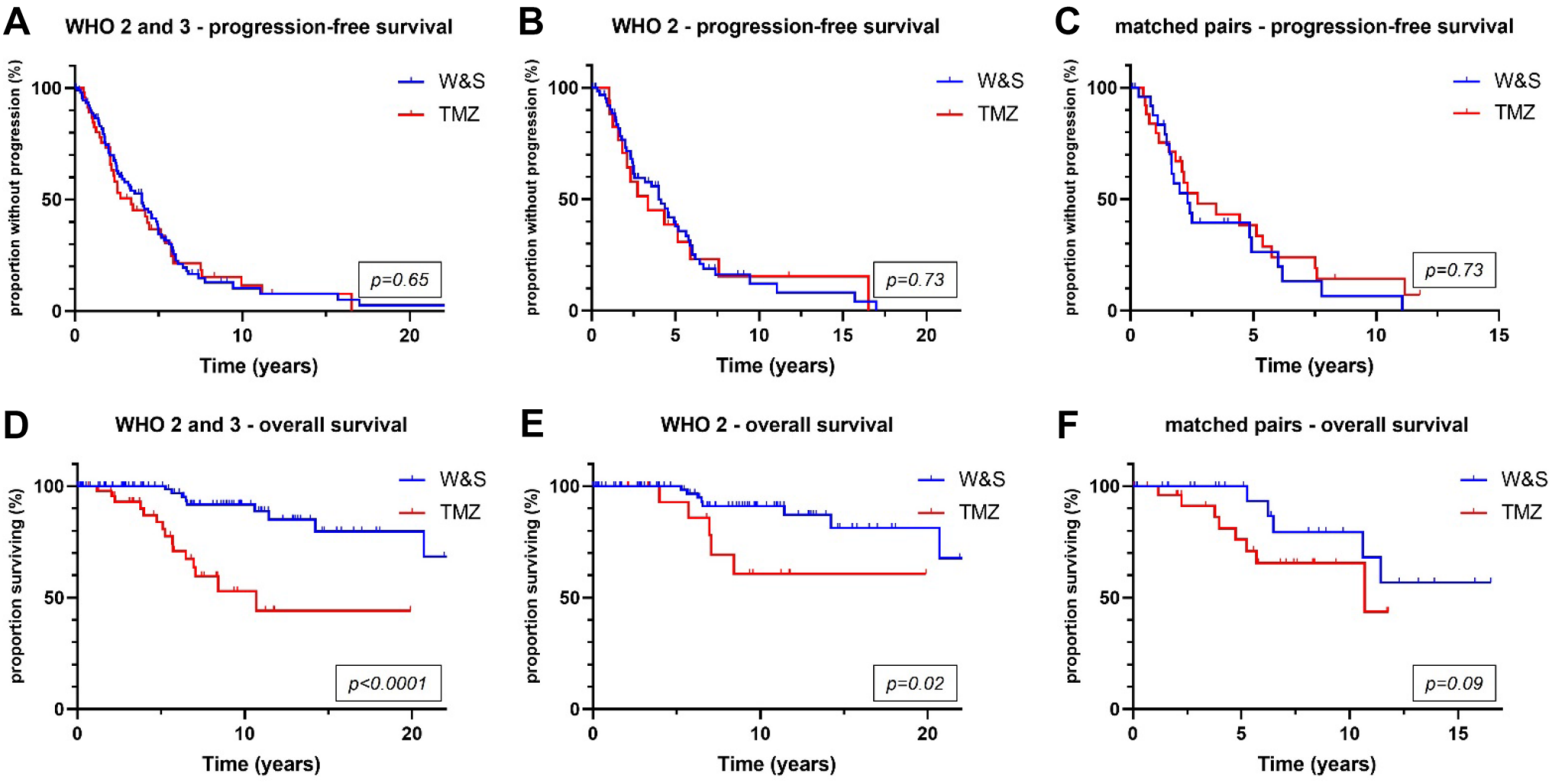
Wait-and-scan strategies and temozolomide chemotherapy showed similar progression-free survival (**A**–**C**). Waitand-scan strategies were associated with longer overall survival (p < 0.01) and in WHO grade 2 gliomas (p = 0.02) (**D**, **E**). In a matched-pair analysis accounting for post-surgical T2 tumor volume and WHO grade, there was an association with prolonged overall survival by trend (p = 0.09) (**F**). WHO World Health Organization; W&S wait-andscan; TMZ temozolomide

PFS was longer in patients treated post-surgically with RT alone than in patients treated with TMZ alone (6.2 vs 3.4 years, *p* = 0.02) and patients monitored by W&S strategies (6.2 vs 4 years, *p* = 0.03) (Fig. 2A). Superiority of RT over TMZ in terms of PFS was confirmed in a matched-pair analysis that comprised 16 pairs (7.8 vs 2.8 years, *p* = 0.03) (Fig. 3A). OS was significantly longer in the RT cohort than in the TMZ cohort (14.4 vs 10.7 years, *p* = 0.02), but not in the matched-pair analysis (*p* = 0.27; number of pairs/events: 8/16) (Figs. 2C, 3B). Comparing W&S with TMZ, patients treated with TMZ alone showed similar PFS, but significantly shorter survival times overall (not reached vs 10.7 years, *p* < 0.01) and in a subgroup analysis of WHO grade 2 astrocytomas only (*p* = 0.02) (Fig. 4D, E). Patients with WHO grade 3 astrocytomas receiving radiotherapy lived significantly longer than patients treated with temozolomide (14.3 vs 10.7 years, p = 0.01). For WHO grade 3 gliomas, there was an association by trend between longer overall survival and wait-and-scan strategies as opposed to temozolomide (18.3 vs 10.7, p = 0.08).

**Fig. 5:**
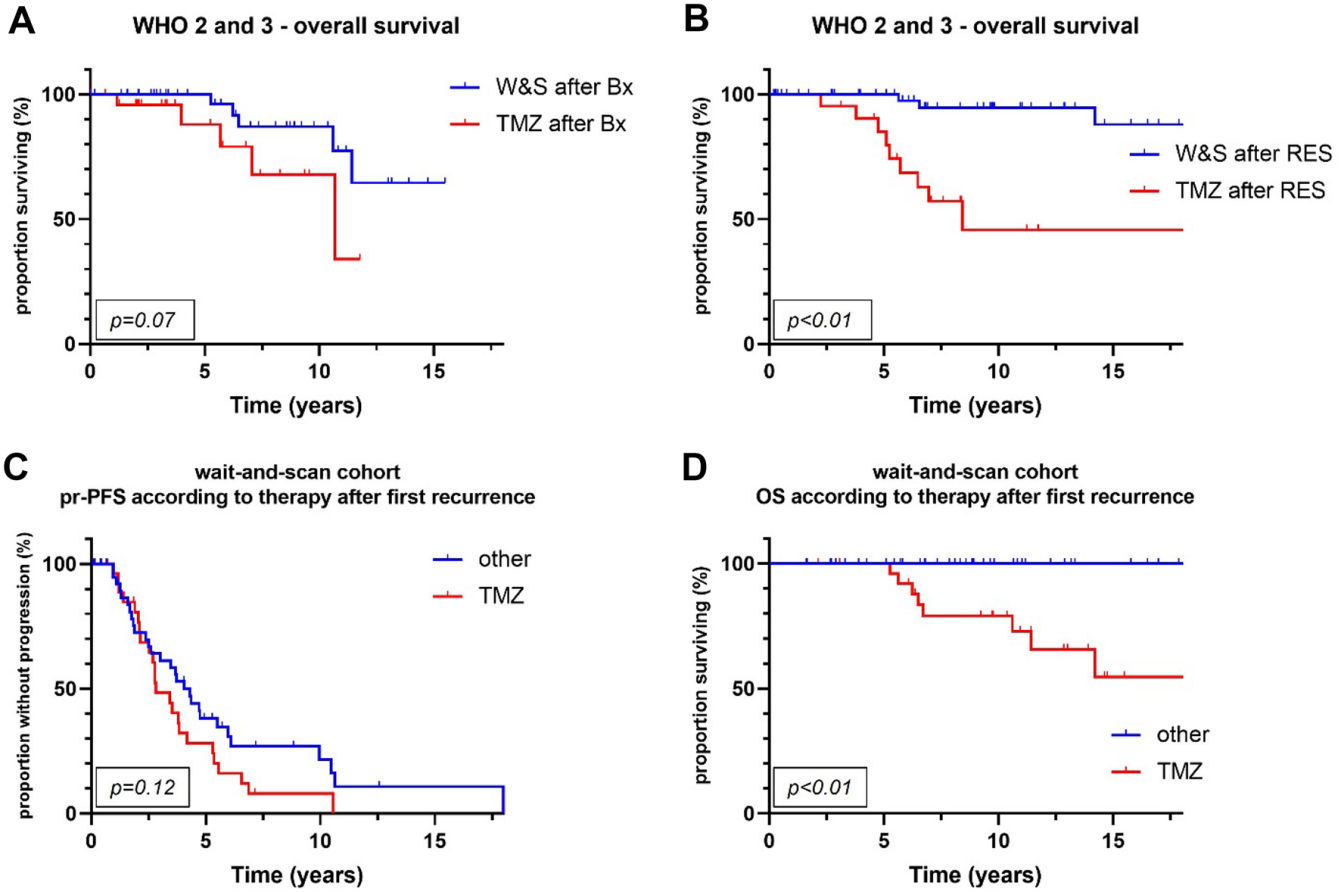
Wait-and-scan strategies after stereotactic biopsy were associated with longer overall survival than temozolomide alone after stereotactic biopsy by trend (p = 0.07) (**A**). Patients monitored by means of wait-and-scan strategies after tumor resection showed significantly longer overall survival times than patients treated with temozolomide alone after tumor resection (p < 0.01) (**B**). Patients initially monitored by active surveillance (wait-and-scan cohort) and experiencing tumor progression were further stratified according to the initiated therapy at first recurrence. There was no significant difference in progression-free survival between patients treated with temozolomide alone and any other treatment strategy at first progression (p = 0.12) (**C**). Temozolomide alone at first recurrence was associated with shorter OS (p < 0.01) (**D**). *WHO* World Health Organization; *W&S* wait-and-scan; *Bx* stereotactic biopsy; *TMZ* temozolomide; *RES* tumor resection; *pr-PFS* post-recurrence progression-free survival

In a matched pair analysis, in which 28 pairs were identified, TMZ was associated with poorer survival by trend (*p* = 0.09) when compared to W&S strategies (Fig. 4F). In a subgroup analysis according to the mode of initial histological sampling, there was an association between longer overall survival and W&S strategies after biopsy as opposed to TMZ alone after biopsy by trend (p = 0.07) (Fig. 5A). This difference was significant in patients initially receiving tumor resection (p < 0.01) (Fig. 5B). Patients with tumor progression from the W&S cohort were stratified according to the first treatment after progression. Here, TMZ alone was associated with shorter OS than other treatments (p < 0.01), although mean tumor volumes were smaller in the TMZ subgroup with 46 cm^3^ versus 54 cm^3^ (Fig. 5D).

In addition to the Kaplan–Meier estimates determined for the different treatment cohorts, several variables commonly associated with PFS and OS were tested in univariate analyses. None of the tested variables (patient age at diagnosis, sex, CNS WHO grade at diagnosis, KPS, post-surgical T2 volume, contrast enhancement on initial MRI, MGMT promoter methylation) were associated with PFS (Table 2). CNS WHO grade, post-surgical T2 tumor volume and contrast enhancement on initial MRI, however, were associated with OS (Table 2). Multivariate analysis for OS did not show significant associations, but the number of events was low with 33 tumor-related deaths, limiting the interpretation (Table 3).

**Table 2:** Univariate analysis of patient-related factors potentially associated with progression-free survival or overall survival

Factor	PFS			OS		
	HR	95% CI	*p* value	HR	95% CI	*p* value
Age^x^	0.99	0.97–1.02	0.64	1.0	0.97–1.03	0.94
Sex, female vs male	1.35	0.75–2.47	0.33	1.14	0.56–2.33	0.72
KPS^x^	1.01	0.94–1.09	0.9	0.98	0.89–1.08	0.63
Post-surgical T2 tumor volume^x^	1.00	0.99–1.01	0.86	1.01	1.00–1.02	< 0.01
CE on MRI yes vs no	1.38	0.93–2.03	0.11	2.81	1.30–6.29	< 0.01
Postsurgical strategy (TMZ vs W&S vs RT)	0.83	0.38–1.68	0.62	6.78	2.87–17.25	< 0.01
CNS WHO grade 3 vs 2	1.16	0.63–2.21	0.65	3.23	1.59–6.63	< 0.01
MGMT promoter status	0.45	0.19–1.33	0.10	0.86	0.25–5.93	0.84

*Vs* versus; *KPS* Karnofsky Performance Status; *CNS* central nervous system; *WHO* World Health Organization; *MGMT* *O*6-methylguanine-DNA methyltransferase; *CE* contrast enhancement; *MRI*, magnetic resonance imaging, *PFS* progression-free survival; *OS* overall survival; *W&S* wait-and-scan; *TMZ* temozolomide; *RT* radiotherapy

^x^Continuously scaled

**Table 3:** Multivariate analysis of factors showing significant associations with overall survival in univariate analyses

Factor	OS		
	HR	95% CI	*p* value
Post-surgical T2 tumor volume^x^	1.01	1.00–1.02	0.07
CE on MRI yes vs no	1.71	0.65–4.62	0.28
Postsurgical strategy (TMZ vs W&S vs RT)	2.02	0.46–8.76	0.36
CNS WHO grade 3 vs 2	0.38	0.10–1.26	0.14

*Vs* versus; *CE* contrast enhancement; *MRI* magnetic resonance imaging; *OS* overall survival; *TMZ* temozolomide; *W&S* wait-and-scan; *RT* radiotherapy; *CNS* central nervous system; *WHO* World Health Organization

^x^Continuously scaled

## Discussion

Patients with lower grade IDHmut gliomas are often subjected to repetitive treatment over time due to frequent recurrences at a relatively young age [1, 23]. The roles of W&S strategies and chemotherapy regimens alone, e.g., to defer RT, have not been determined conclusively. Especially studies addressing TMZ chemotherapy alone have yielded controversial results, some of which imply potential negative effects on IDHmut tumors due to induction of hypermutation [14–17].

We here present a retrospective outcome analysis of 183 patients with WHO grade 2 or 3 IDHmut astrocytomas initially monitored through a W&S strategy or treated with TMZ alone or RT alone. We accounted for WHO tumor grade, postoperative T2 tumor volumes and contrast enhancement and report superiority of RT over TMZ in terms of PFS and OS. Furthermore, patients initially treated with TMZ showed significantly shorter survival times than patients initially monitored through W&S alone despite identical PFS. This association was observed in multiple subgroup analyses (Figs. 3, 4, 5). Of note, the latter analysis was subjected to substantial selection bias because of significant differences in tumor volume, contrast enhancement and WHO grades between groups that could only be in part accounted for in our analyses (Fig. 3).

Prospective trials respecting the contemporary, molecular classification of adult gliomas and investigating surveillance or monotherapy strategies are scarce. The NOA-04 randomized phase 3 trial investigated RT alone versus chemotherapy alone with either PCV or TMZ in anaplastic gliomas [10, 11]. Here, no differential benefit of RT alone versus chemotherapy alone was reported. In the subgroup of gliomas with 1p/19q-codeletion, i.e., oligodendrogliomas, and a CpG island methylator phenotype, TMZ was inferior to RT and PCV in terms of PFS and there was a trend towards shorter time-to-treatment failure and overall survival [11]. Superiority of RT over TMZ alone regarding PFS in the treatment of IDHmut low-grade astrocytomas was reported in the prospective, phase 3 EORTC 22033-26033 study [9]. The prospective EORTC 22845 randomized trial compared early RT versus RT that was delayed to the timepoint of first progression in lower grade gliomas. The study demonstrated prolonged PFS in the RT cohort, but no significant improvement in OS [6]. These results were mirrored by our data. Prospective trials comparing W&S strategies with chemotherapy alone within the framework of molecularly defined gliomas have yet to be conducted. Whereas the question of whether any of these strategies is superior to the others remains unanswered, clinical trials have demonstrated benefit of combination therapy of RT and alkylating chemotherapy over monomodality treatment in the treatment of IDHmut gliomas. For IDHmut astrocytomas WHO grade 2, radiochemotherapy with sequential PCV after radiation therapy is recommended in older patients or those with residual, posteroperative tumor. This recommendation is based on the RTOG 9802 study that demonstrated prolongated PFS and OS in the radiochemotherapy cohort when compared to RT alone in a large, prospective cohort of WHO grade 2 gliomas [2]. Therapy for WHO grade 3 astrocytomas is mainly determined by the CATNON trial (EORTC study 26053-22054) and consists of RT followed by 12 cycles of maintenance TMZ [24]. Clinical trials investigating RT followed by TMZ versus RT followed by PCV in IDHmut astrocytomas have not been conducted so far.

Despite the positive results from the CATNON study, recently published studies have slightly tempered expectations from TMZ therapy in IDHmut gliomas. Barthel et al. described hypermutator phenotypes after treatment with alkylating agents in diffuse gliomas [25]. In 2020, Touat et al. published sequencing data, analyzing mutational burden in 10′249 gliomas. Here, a therapy-driven induction of hypermutation through acquisition of a mismatch repair deficiency after TMZ therapy was described [15]. Yu et al. sequenced recurrent IDHmut gliomas and confirmed hypermutation in some patients treated with TMZ. They also reported an association with shorter survival and discontiguous, recurrent disease in patients previously treated with TMZ [16]. Still, these data do not warrant a change of current guidelines. The data provided here and within the framework of recent studies investigating TMZ-induced hypermutation, does, however, raise the question whether TMZ is the right choice in patients deemed not eligible for RT and believed to show a long, relatively good clinical course. PCV chemotherapy, or even PC regimens omitting vincristine to limit side effects, might be appropriate alternatives if W&S strategies are not justifiable. Multiple studies have shown efficacy of PCV chemotherapy after RT [2, 3, 26]. Data on surveillance strategies cannot be expected soon. The wait-or-treat study (IWOT; NCT03763422) sought to investigate W&S strategies versus radiochemotherapy after gross total resection, but was closed prematurely because of poor accrual. Chemotherapy alone is not the gold standard in the treatment of IDHmut gliomas, but there will be patient subgroups not eligible for standard therapy with RT, e.g., due to extensive tumor volumes. These patients are subjected to a selection bias and will have to be treated based on recommendations supported by comparably low levels of evidence. Prospective studies on monochemotherapy are not likely to be initiated in the future.

The major shortcoming of this study is its retrospective nature and thus selection bias. Some patient characteristics differed significantly between the cohorts. In the RT cohort, patients were older at diagnosis. In the TMZ cohort, clinical status was worse and more importantly, post-surgical T2 tumor volumes were larger. The proportion of WHO grade 2 as opposed to grade 3 astrocytomas was higher in the W&S cohort, favouring this cohort. These differences were in part corrected for through matched-pair and subgroup analyses (Figs. 3, 4).

Conclusions from retrospective data must be drawn with caution, but our data within the framework of recent studies favor RT over TMZ alone and justify a critical view on initial TMZ monotherapy for IDHmut astrocytomas [15, 16, 25]. The results on RT match prospective data. Even though the TMZ cohort is at a disadvantage, especially because of the comparably large tumor volumes, the inferiority as compared to the W&S cohort in terms of survival despite identical initial PFS was clear and highly significant. The fact that there was no difference in PFS might point towards a long-term effect of potential genetic alteration through TMZ therapy. This might not become clinically relevant in more aggressive, high-risk gliomas or glioblastomas. If IDHmut astrocytomas previously treated with TMZ recur, caretakers might consider conducting a biopsy if the patient is not eligible for tumor resection to investigate mutational burden and potentially find molecular alterations that allow for targeted therapy [27]. So far, there is only indirect data on whether there is any tumor specific benefit from TMZ monotherapy when compared to no further therapy beyond surgery. Taking the retrospective nature of this study into account, one might at least hope for similar, if not better, clinical outcome in a cohort of patients that had received a median of 6 months of TMZ chemotherapy as compared to no therapy at all after histological sampling.

## Data Availability

Clinical and molecular data on all patients are anonymized and stored in local data bases secured by passwords.

## Declarations

### Research involving humans and/or animals participants

The present study was conducted retrospectively.
